# Antimicrobial skin peptides in premature infants: Comparison with term infants and impact of perinatal factors

**DOI:** 10.3389/fimmu.2023.1093340

**Published:** 2023-02-27

**Authors:** Alexander Humberg, Lisa Neuenburg, Hannah Boeckel, Mats Ingmar Fortmann, Christoph Härtel, Egbert Herting, Heilwig Hinrichs, Franziska Rademacher, Jürgen Harder

**Affiliations:** ^1^ Department of General Pediatrics, University Children’s Hospital Muenster, Muenster, Germany; ^2^ Institute of Medical Biometry and Statistics, University of Luebeck, Luebeck, Germany; ^3^ Department of Pediatrics, University Hospital Schleswig-Holstein, Lübeck, Germany; ^4^ Department of Pediatrics, University Hospital, Wuerzburg, Germany; ^5^ Department of Dermatology, Venerology and Allergology, Quincke Research Center, Kiel University, Kiel, Germany

**Keywords:** antimicrobial peptides, premature infants, sepsis, skin barrier, chorioamnionitis

## Abstract

**Introduction:**

Preterm infants have an immature epidermis barrier function that may lead to an increased permeability to pathogens. On the surface of the human skin, antimicrobial peptides (AMPs) are important molecules of the innate immune system, have broad antimicrobial properties, and provide an essential role in integrity of the microbiome. Given the marked susceptibility of preterm infants to infection, we hypothesize a decreased expression of AMPs on the skin of preterm infants.

**Materials and methods:**

In a prospective single-center study with 35 preterm and 20 term infants, we analyzed skin rinsing probes for the presence of the AMPs psoriasin (S100A7) and ribonuclease 7 (RNase 7) *via* enzyme-linked immunosorbent assay. Samples were taken from preterm infants < 34 0/7 weeks gestational age (mean ± SD gestational age, 28.8 ± 2.4 weeks) on days 0, 7, 14, and 28 after birth. Term infants (> 36 6/7 weeks) (controls) were washed on days 0 and 28.

**Results:**

Psoriasin and RNase 7 were both expressed on skin of preterm and term infants and increased in concentration significantly over time. RNase 7 was more expressed in term infants on day 0 [preterm = 1.1 (0.7–2.9) vs. term = 2.0 (1.1–3.4) ng/ml, p = 0.017]. On day 28, premature infants showed higher values of psoriasin [preterm = 10.9 (5.6–14.2) vs. term = 6.3 (3.4–9.0) ng/ml, p < 0.001]. Notably, preterm infants with infectious or inflammatory context driven by histological proof of chorioamnionitis and early-onset or late-onset sepsis had higher concentrations of psoriasin as compared with non-affected preterm infants. After exclusion of infants with inflammatory hit, median concentrations of RNase 7 and psoriasin did not differ between preterm and full-term infants on days 0 and 28.

**Discussion:**

Psoriasin and RNase 7 concentrations increase over time on the skin of newborn infants and seem to play a role in the first defense against infection. This is of particularly interest as the role of AMPs on a maturing skin microbiome and its possible new prevention strategies is unclear and needs to be determined.

## Introduction

Antimicrobial peptides (AMPs) on the surface of the skin have broad antimicrobial properties and contribute to immune protection as first line innate immune barrier (1, 2). In addition, AMPs play an essential role in establishing and maintaining a homogeneous colonization of the skin with microorganisms (3). AMPs are primarily released by cells of the immune system but are also produced by epithelial cells within the skin and at mucosal surfaces (4–7). Defensins [human beta-defensin (hBD)], cathelicidins (LL-37), psoriasin, ribonuclease 7 (RNAse 7), dermcidin, and adrenomedullin have been described on human skin. Because of their broad antimicrobial properties against bacteria, viruses, and fungi, these peptides enable effective immune protection. RNase 7 is able to prevent colonization by S. aureus and exhibit high antimicrobial activity in vitro against a variety of gram-positive and gram-negative bacteria and yeasts (8). The antimicrobial activity of psoriasin against pathogens is mediated by zinc deprivation. In healthy skin, psoriasin is continuously expressed and released in high concentrations and, therefore, effectively protects the skin from colonization and infection by E. coli. It shows increased expression after stimulation with bacteria or proinflammatory cytokines such as interleukin-1 (IL-1) or IL-17 (9).

Preterm infants born before completion of 36 weeks’ gestation are at increased risk for neonatal infections with significant effects on mortality and long-term morbidity (10, 11). Multiple factors and, mainly, a dysregulation of the immune system significantly contribute to an increased infection risk [for review, see (11)]. The skin of preterm infants has a reduced barrier function as the stratum corneum (SC) of the epidermis has fewer layers, vernix caseosa is not present, and reduced integrity function in the first weeks of life renders preterm skin permeable to pathogens. Furthermore, injuries of the premature skin through vascular access devices or adhesives play a key role for nosocomial infections (11).

It was found that, in relation to term infants, preterm infants demonstrate lower levels of AMPs in plasma, lung lavage, and feces (12–15). Furthermore, the expression of AMPs in the fetal skin depends on the maturity of the skin and the innate immune system (16). The deficiency of AMPs in early life may contribute to an increased risk for invasive infections. However, no data exist about AMP concentrations of the skin of premature infants and their role in immune protection of these vulnerable infants.

We here aim to characterize the expression and composition of skin AMPs in preterm and term infants in a longitudinal fashion within the first 28 days of life. We documented clinical factors that might influence the expression of AMPs, i.e., gestational age, histological chorioamnionitis, neonatal sepsis, and preterm skin care with sunflower oil. We addressed the expression of psoriasin (S100A7) and RNase 7 as most abundant AMPs on human skin surface (7).

## Materials and methods

### Study cohort

All infants were born and recruited at the Department of Pediatrics, University Hospital Schleswig-Holstein, Campus Lübeck, between November 2019 and March 2021. Immediately after birth, infants were evaluated for inclusion criteria. To control for premature effects on AMP concentrations, we recruited infants with gestational age of < 34 0/7 weeks and as control infants > 36 6/7 weeks gestational age. Infants with diseases or injuries of the skin were not included. In term infants, any externals or cosmetics had to be left out. As most preterm infants received sunflower oil treatment of the skin, the left upper thigh was left out to control for effects of sunflower oil treatment. To reduce inter-observer variability, all patients are assessed only by two different researchers (LN and HB).

### Sampling and quantitative determination of AMPs

From all participants, non-invasive skin wash probes were taken from different sites (abdomen, left and right thigh, and upper arm). Skin rinsing procedure was performed in preterm infants immediately after birth on days 0, 7, 14, and 28 and in term infants on days 0 and 28. The skin was flushed with 1 ml of 37°C warmed buffer solution [10 mM sodium phosphate buffer (pH 7.2) with 0.1% Triton X-100] through sterile plastic tubes by pipetting 10 times up and down. The liquid was subsequently stored in tubes and immediately centrifuged (10 min, 10,000×g) and diluted 1:10 with buffer solution containing 10% (w/v) bovine serum albumin, aliquoted, and stored at −80°C. All materials are sent for laboratory analysis to the Department of Dermatology, University Hospital Schleswig-Holstein, Campus Kiel. After centrifugation, supernatants were analyzed for AMPs *via* “enzyme linked immunosorbent assay” (ELISA) as described elsewhere (17, 18). To analyze psoriasin expression, a monoclonal antibody from hybridoma mouse cells was used. For RNase 7 detection, the polyclonal antibody was derived from goat [23].

Placenta specimen from infants born < 34 0/7 weeks gestational age were analyzed for presence of histological chorioamnionitis at the Institute of Pathology, University Hospital Schleswig-Holstein, Campus Lübeck.

### Definitions

Gestational age was calculated from the best obstetric estimate based on early prenatal ultrasound and obstetric examination. Sepsis was defined as condition when neonatologists decided to treat the infant with antibiotics and to continue for at least 5 days due to the following reasons: ≥ 2 clinical signs of systemic inflammatory response: temperature > 38°C or < 36.5°C, tachycardia > 200/min, new onset or increased frequency of bradycardias or apneas, hyperglycemia > 140 mg/dl, base excess < −10 mval/l, changed skin color, and increased oxygen need; and 1 laboratory sign: C-reactive protein > 10mg/L, platelet count < 100/nl, immature/total neutrophil ratio > 0.2, and white blood cell count < 5/nl (NeoKISS) (19). Early-onset sepsis (EOS) was defined as signs of sepsis within the first 72 h after birth with or without proof of a causative agent in blood culture. Late-onset sepsis (LOS) was defined as signs of sepsis after the first 72 h after birth with or without proof of a causative agent in blood culture. Small for gestational age is defined as birth percentile < 10th according to gestational age (20).

### Ethical approval

Approval by the local ethics committee for research in human subjects of the University of Lübeck (file number 19-028) has been granted. The study protocol was registered on the German Clinical Trials Register portal (DRKS00021635) on 12 May 2020. The study is compliant with the Health Insurance Portability and Accountability Act of 1996 (HIPAA). The work has been carried out in accordance with The Code of Ethics of the World Medical Association (Declaration of Helsinki) for experiments involving humans. Immediately after birth and stabilization of the infants, oral consent was taken from parents or legal guardians prior the first skin washing. Within 48 h after birth, all parents or legal guardians were contacted for written informed consent. If then parents refused to participate, all materials and data were disposed.

### Data collection and statistical analysis

Medical data were collected, pseudonymized, and entered into a data base. Clinical data concerning perinatal information, infections, antibiotic therapy duration, and duration of phototherapy were recorded.

Values of different probe sites (abdomen, thigh, and arm) were pooled and analyzed. Data were presented as means with standard deviation (SD), medians with interquartile ranges (IQRs), as well as numbers with frequency (n%) and their corresponding 95% confidence intervals (CIs). Differences of independent groups are tested *via* Mann–Whitney U-test and Kruskal–Wallis test. Wilcoxon signed-rank test is used to compare two related samples. Ordinary one-way ANOVA with Holm–Sidak’s multiple comparisons test was used for comparison of more than three related samples. The level of significance is set at p < 0.05.

Intra-assay coefficient of variation (CV) and inter-assay CV was calculated within each plate and the average CV across all plates. CV% was calculated by dividing the SDs by the means and converting to a percentage (×100).

All statistical analyses were performed with SPSS 26.0 software (IBM SPSS Statistics for Windows, Version 27.0. Munich, Germany) and with GraphPad Prism (Version 7.0, San Diego, CA).

### Financial disclosure

This project was financed with funds from the Department of Pediatrics, University Hospital of Schleswig-Holstein, Campus Lübeck and the Department of Dermatology, Venerology and Allergology, Quincke Research Center, University-Hospital Schleswig-Holstein, Campus Kiel.

## Results

### Study cohort

**Figure 1:**
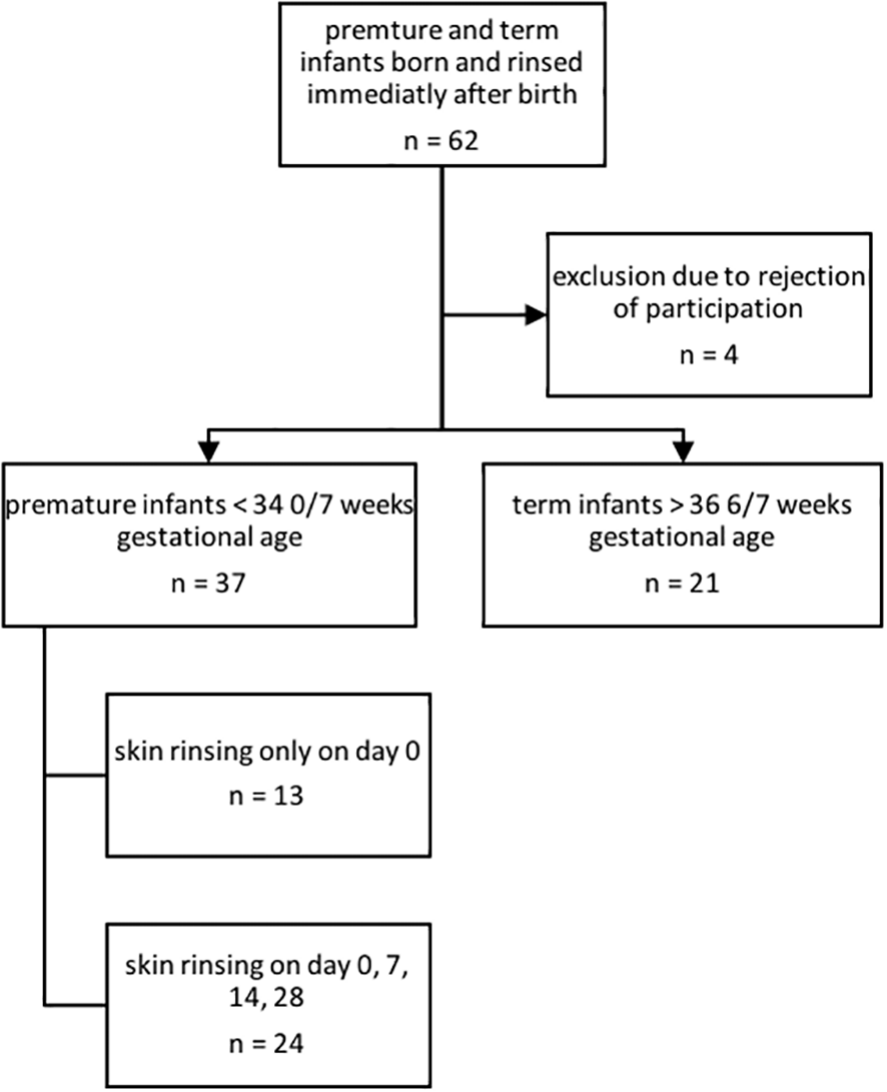
Inclusion and exclusion of premature and term infants for AMP characterization.

In this convenience sample study, a total of n = 62 infants had a first skin rinsing probe taken on day 0 of life (see **Figure 1**).

After refusal of participation by parents of four infants and exclusion due to unmet inclusion criteria (gestational age out of scope), n = 55 infants consisting of n = 35 premature infants and n = 20 term infants were included and followed up for 28 days. For further baseline cohort characteristics, please see **Table 1**. Until corrections of the study schedule, in n = 13 premature infants, rinsing solution was only collected on the first day of life.

**Table 1:** Baseline characteristics of included preterm and term infants.

Characteristics	Preterm infants (< 34 1/7 weeks gestational age)	Term infants (> 36 6/7 weeks gestational age)
	n = 35	n = 20
Gestational age (weeks)	28.8 (2.4)	39.5 (1.2)
Birth weight (grams)	1214 (508)	3454 (490)
Vaginal delivery	3 (8.6)	14 (70.0)
Caesarean section	32 (91.4)	6 (30.0)
Retinopathy of praematorum (therapy)	4 (11.4)	n.a.
Intraventricular hemorrhage	5 (14.3)	n.a.
Bronchopulmonary disease	7 (20.0)	n.a.
Small for gestational age	7 (20.0)	n.a.
Maternal antibiotics	31 (88.6)	1 (25.0)
Chorioamnionitis	12 (34.3)	n.a.
Early-onset sepsis	8 (22.9)	2 (10.0)
Late-onset sepsis	11 (31.4)	n.a.
Antibiotic therapy of infant	22 (62.9)	4 (20.0)

Data are given as mean (SD) or n (%) with column percentages. n.a., not applicable.

The inter-assay CV for the psoriasin ELISA was 8.1%, and the intra-assay CV was 8.5%. For RNase 7, the inter-assay CV was 6.9%, and the intra-assay CV was 8.1%.

### Lower psoriasin levels on day 0 and higher RNase 7 levels on day 28 in preterm infants

**Figure 2:**
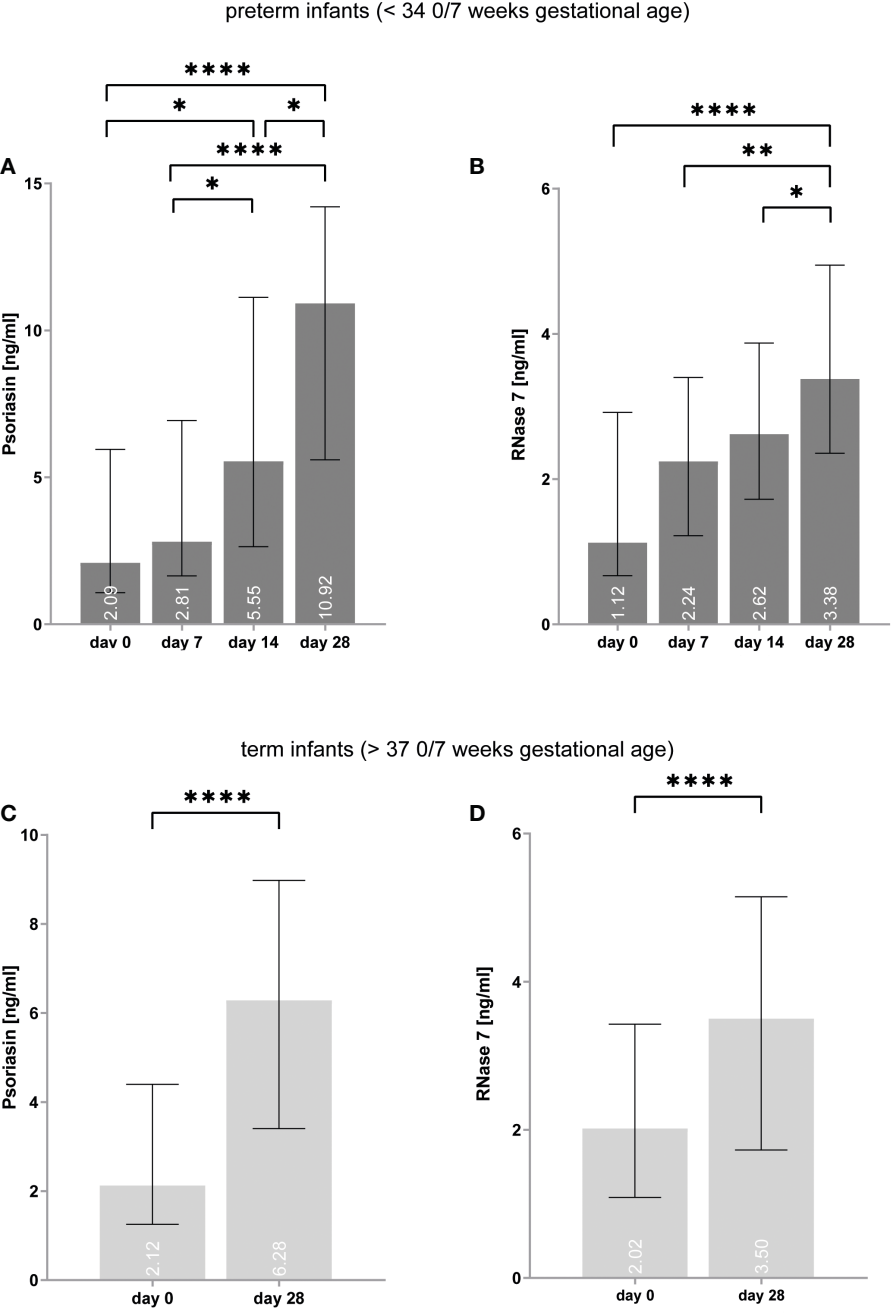
**(A–D)** Expression of psoriasin and RNase 7 in premature **(A, B)** and term **(C, D)** infants increases over time. *p < 0.05, **p < 0.01, and ****p < 0.0001 (ordinary one-way ANOVA with Holm–Sidak’s multiple comparisons test for preterm infants, Wilcoxon signed-rank test for term infants). Medians are given for each day in white numbers.

On day 0, the median expression of psoriasin was similar in both preterm and term infants [preterm = 2.1 (IQR: 1.1–5.9) vs. term = 2.1 (1.3–4.4) ng/ml, p = 0.320], but RNase 7 was more expressed in term infants on day 0 [premature = 1.1 (0.7–2.9) vs. term = 2.0 (1.1–3.4) ng/ml, p = 0.017]. On day 28, premature infants showed higher values of psoriasin [preterm = 10.9 (5.6–14.2) vs. term = 6.3 (3.4–9.0) ng/ml, p < 0.001], but not for RNase 7 [preterm = 3.4 (2.4–4.9) *vs*. term = 3.5 (1.7–5.1) ng/ml, p = 0.574] (see **Figure 2**).

### The concentrations of psoriasin and RNase 7 increase over time in preterm and term infants

In preterm infants, psoriasin concentration was found to elevate from median values of 2.1 (1.1–5.9) ng/ml on day 0 to 10.9 (5.6–14.2) ng/ml on day 28 (p < 0.0001) (see **Figure 2**). In term infants, psoriasin increased from 2.1 (1.3–4.4) ng/ml to 6.3 (3.4–9.0) ng/ml (p < 0.0001). RNase 7 was also found to have higher concentrations on day 28 with 1.1 (0.7–2.9) ng/ml on day 0 and 3.4 (2.4–4.9) ng/ml on day 28 in premature infants (p < 0.0001) as well in term infants [2.0 (1.1–3.4) vs. 3.5 (1.7–5.1) ng/ml, p < 0.0001].

### Presence of histological chorioamnionitis increases expression of psoriasin

**Figure 3:**
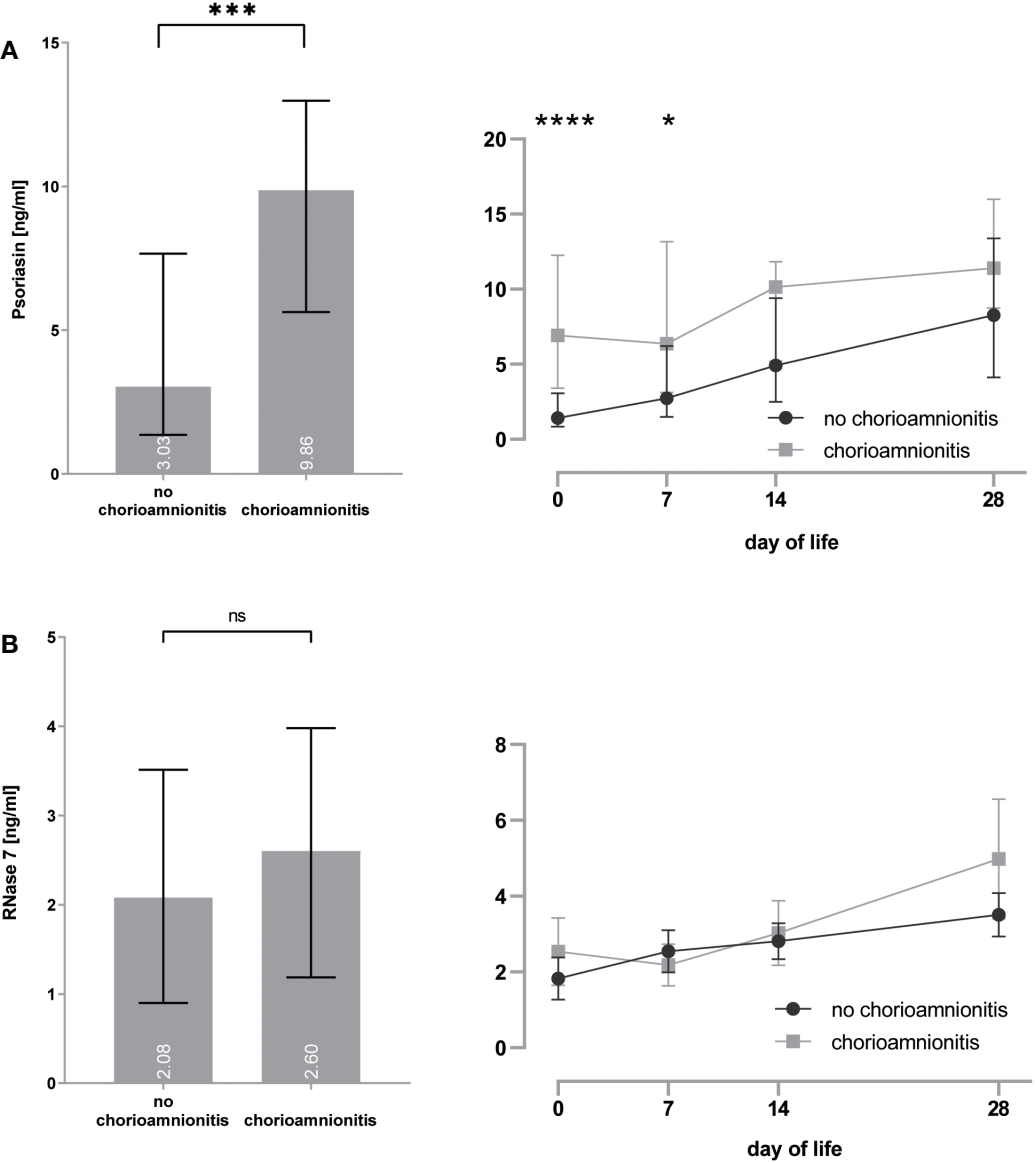
**(A, B)** Premature infants with histological proof of chorioamnionitis show increased concentrations of psoriasin **(A)**, but no differences for RNase 7 **(B)** when compared to infants without signs of chorioamnionitis. *p < 0.05, ***p < 0.001, and ****p < 0.0001 (Mann–Whitney U-test for left bar graphs, Kruskal–Wallis Test for right line diagram). Medians are given for each day in white numbers. ns, not significant; p > 0.05.

*In utero* exposure to placental inflammation, i.e., histological chorioamnionitis, increased the psoriasin [3.0 (1.4–7.7) vs. 9.9 (5.6–13.0) ng/ml, p < 0.0001] expression on the skin of preterm infants (see **Figure 3**), particularly for day 0 [1.4 (0.8–3.1) vs. 6.9 (3.4–12.3) ng/ml, p < 0.0001] and day 7 [2.7 (1.5–6.2) vs. 6.3 (3.1–13.2) ng/ml, p = 0.014] after birth. No significant effect was noted for the RNase 7 [2.1 (0.9–3.5) vs. 2.6 (1.2–4.0) ng/ml, p = 0.058] expression.

### Sepsis influences expression of psoriasin and RNase 7

**Figure 4:**
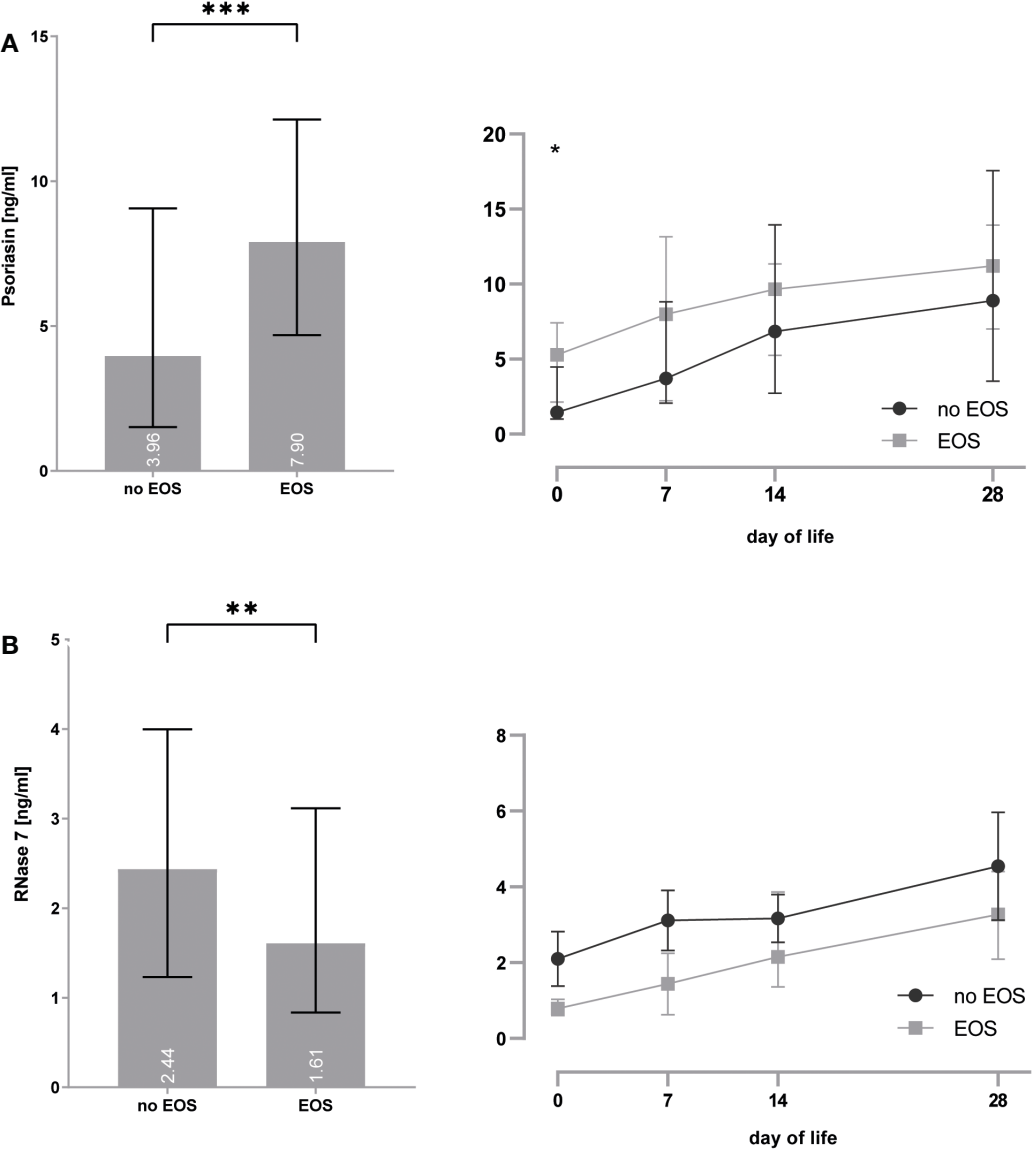
**(A, B)** Premature infants with clinical signs of early-onset sepsis (EOS) show increased concentrations of psoriasin **(A)** and decreased concentrations of RNase 7 **(B)** when compared to infants without signs of EOS. *p < 0.05, **p < 0.01, and ***p < 0.001 (Mann–Whitney U-test for left bar graphs, Kruskal–Wallis Test for right line diagram). Medians are given for each day in white numbers.Infants with LOS had increased expression of psoriasin [2.6 (1.5–6.5) vs. 7.4 (2.7–11.7), p < 0.0001], especially on day 7 [2.0 (1.3–3.6) vs. 7.6 (2.8–10.9), p = 0.008], but no differences in the concentrations of RNase 7 were found (see **Figure 5**).

Preterm infants with EOS showed increased psoriasin concentrations [4.0 (1.5–9.1) vs. 7.9 (4.7–12.1) ng/ml, p = 0.0002] particularly on day 0 [1.5 (1.0–4.5) vs. 5.3 (2.1–7.4) ng/ml, p = 0.014] and decreased RNase 7 concentrations [2.4 (1.2–4.0) vs. 1.6 (0.8–3.1), p = 0.01] (see **Figure 4**).

**Figure 5:**
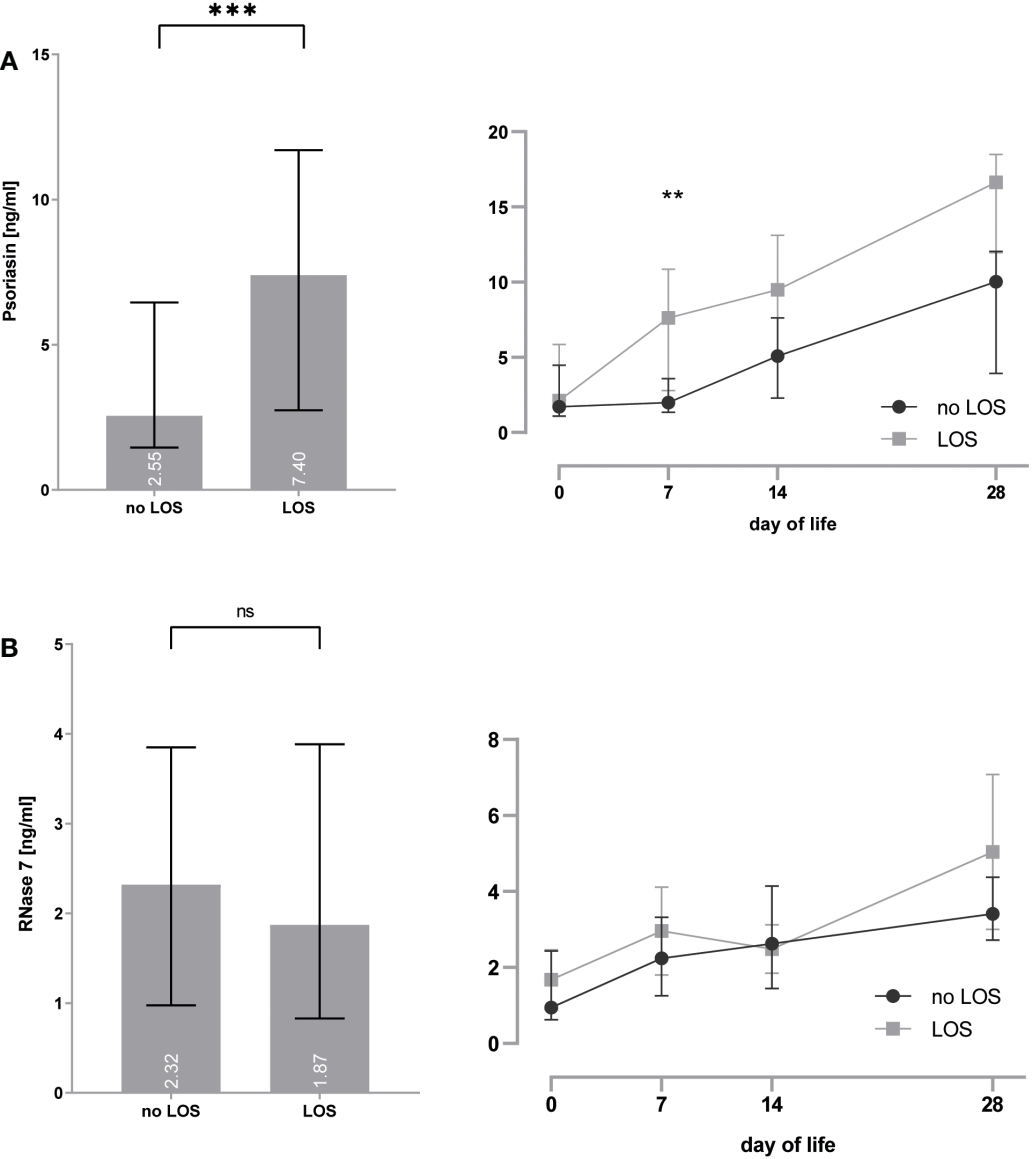
**(A, B)** Premature infants with clinical signs of late-onset sepsis (LOS) show increased concentrations of psoriasin **(A)**, but no differences for RNase 7 **(B)** when compared to infants without signs of LOS. **p < 0.01 and ***p < 0.0001 (Mann–Whitney U-test for left bar graphs, Kruskal–Wallis Test for right line diagram). Medians are given for each day in white numbers. ns, not significant; p > 0.05.

### Oil skin care does not influence expression of psoriasin and RNase 7

Left and right upper thigh indicating use of sunflower oil for skin care in preterm infants with (right) and without (left upper thigh) care did not show significant differences for AMP expression (**Table 2**).

**Table 2:** Median (IQR) of psoriasin and RNase 7 concentrations of different probe sites and different days in preterm infants.

Day	Psoriasin		p-values	RNase 7		p-values
	Thigh right	Thigh left		Thigh right	Thigh left	
0	2.1 (0.9–5.7)	1.4 (0.8–4.5)	0.003	1.6 (0.8–3.1)	1.4 (0.7–3.6)	0.554
7	2.2 (1.3–2.9)	2.7 (1.3–4.7)	0.526	2.5 (2.2–3.5)	2.5 (1.5–3.9)	0.375
14	3.6 (2.6–5.1)	4.0 (1.7–7.8)	0.868	3.1 (1.3–4.4)	2.0 (1.4–3.8)	0.394
28	11.2 (5.8–16.7)	10.7 (6.3–14.1)	0.375	3.5 (2.4–7.0)	3.3 (2.1–5.3)	0.339

P-values derived from the Wilcoxon test. Right thigh was regularly cared for with sunflower oil, and left thigh was left out.

### Expression of psoriasin and RNase 7 in premature infants in absence of chorioamnionitis and/or sepsis

After exclusion of premature infants with proof of chorioamnionitis and sepsis (EOS and LOS), n=14 infants remained for analysis. This subcohort is characterized for later weeks of gestational age [30.1 (2.4) weeks] and increased birth weight [1,423 (643) g]. Here, concentrations of psoriasin and RNase 7 differ between first days of life and day 28 (see **Table 3**). We compared medians of psoriasin and RNase 7 in premature and term infants with exclusion of those with inflammatory complications. No further differences were found concerning the concentrations on days 0 and 28 in both groups (see **Table 4**).

**Table 3:** Median (IQR) concentrations of psoriasin and RNase 7 in premature infants after exclusion of infants with chorioamnionitis and/or sepsis.

Day	Psoriasin	p-values	RNase 7	p-values
0	1.44 (0.84–3.65)	0.036^Δ^	1.63 (1.01–6.24)	
7	1.7 (1.34–4.44)	0.018^Ω^	1.95 (1.82–3.32)	0.028^Ω^
14	3.57 (1.97–10.87)	0.012^Φ^	2.60 (2.50–3.43)	
28	6.75 (2.34–20.69)	0.018^§^	3.59 (1.80–5.93)	0.028^§^

P-values derived from Wilcoxon test and were given for significant findings (p < 0.05). ^§^, day 0 vs. day 28; ^Φ^, day 7 vs. day 14; ^Ω^, day 7 vs. day 28; ^Δ^, day 0 vs. day 14.

**Table 4:** Median (IQR) concentrations of psoriasin and RNase 7 in premature and term infants after exclusion of infants with chorioamnionitis and/or sepsis. P-values derived from Mann–Whitney U-test. No significant findings (p < 0.05).

Day	Psoriasin			RNase 7		
	Premature	Term	p-value	Premature	Term	p-value
0	1.44 (0.84–3.65)	1.92 (1.66–3.56)	0.135	1.63 (1.01–6.24)	2.25 (1.35–2.68)	0.667
28	6.75 (2.34–20.69)	6.45 (5.61–8.68)	0.891	3.59 (1.80–5.93)	4.02 (2.57–5.22)	0.630

## Discussion

In a convenience sample study, we demonstrated for the first time that psoriasin and RNAse 7 levels (a) accelerate expression over time and (b) correlate with chorioamnionitis (psoriasin) or sepsis. We further found that psoriasin and RNAse 7 levels do not differ between preterm and term infants with respect to day of life.

AMPs are essential molecules of the innate and adaptive defense and are expressed on sites with close contact to microorganisms such as the skin. Because of their broad antimicrobial properties against bacteria, viruses, and fungi, these peptides provide effective protection against pathogens but are also believed to shape the preferred microbiota (21). Typical AMP members expressed in human skin are the hBDs, cathelicidin (LL-37), psoriasin, RNase 7, dermcidin, and adrenomedullin (8, 22–26). In vernix caseosa, a substance covering the human fetus from the 28th week of gestation and in the epidermis of fetuses from 18 weeks, AMPs including psoriasin and RNase 7 are detectable (27–29). Because AMP measurement on the vulnerable skin surface of newborns requires the use of non-invasive methods, we used an established skin rinsing method to quantify AMPs on the newborn skin. Skin rinsing is an established method to quantify AMPs and comparable with the tape stripping method (30). Increase of AMP level on the skin surface of patients with inflamed skin (e.g., psoriasis and atopic dermatitis) measured by the skin rinsing method correlates well with increase of AMP immunostainings in biopsies (31). We also measured levels of hBD-2 and hBD-3, which were under the detection limit of the ELISA. In our prospective study, we noted that, with increasing postnatal age, concentrations of psoriasin und RNase 7 rise toward those found on healthy adult skin. The reason for this is unknown but could be assumed as an accompanying effect of AMP development with postnatal epithelial development as the skin structures mature within the first weeks of life (32, 33). Another reason for increasing concentrations after birth could be the contact of the skin to the outer world (29). AMPs play an essential role in maintaining a homogeneous colonization of the skin with microorganisms. For example, Staphylococcus epidermidis is the most frequently isolated species of the skin microbiome and a common pathogen of neonatal LOS (34). There is emerging evidence that the induction of RNase 7 and other skin AMPs is triggered by members of the microbiota such as Staphylococcus epidermidis to shape and balance skin colonization (21). Colonization with bacterial strains starts immediately after birth and could be an important component for increasing AMPs. The increase of AMP expression may prepare the skin to cope with or avoid future infections.

In our study, preterm infants showed higher concentrations of psoriasin than term controls. This observation can be explained by the high rate of inflammatory events such as chorioamnionitis, EOS, and LOS in this vulnerable cohort. Infants with these complications show increased levels of psoriasin. This effect is found at first days of life in infants with chorioamnionitis and EOS and at later days in infants with LOS, indicating a time-dependent inflammatory reaction. AMPs are important effector molecules in the regulation and communication of the innate and adaptive immune system through the control of cell migration, proliferation and differentiation, modulation of toll-like receptors, and the production of cytokines (35–40). Stimuli such as the expression of proinflammatory cytokines, mechanical injuries, or inflammatory processes lead to an upregulation of AMPs (32) and could explain our observation of higher psoriasin levels in premature infants. Total concentrations of RNase 7 were decreased in infants with EOS. This is of particular interest as, for example, in adult patients, reduced RNase 7 concentrations in urines are associated with a higher risk for urinary tract infections (41). This might explain and give a reason why certain populations are more susceptible to infections, which needs to be subject for large-scale prospective studies.

Neonatal sepsis is often the result of infections with bacterial, viral, or fungal microorganisms with a case fatality rate inversely related to gestational age (42). Transmission of pathogens in EOS occurs in utero transplacentar, via ascending bacteria entering the uterus or during the passage of the newborn through the birth canal. Late-onset bloodstream infections occur more frequently in neonates with central venous access or other medical devices. However, the intestines and lungs of especially preterm infants are also an important source of infections.

We could show that preterm and term infants show reduced concentrations of AMPs at the beginning of life but develop similar concentrations on the skin within the first weeks of life. This observation joins many studies on the development of the immune system that is constantly maturing in newborn infants (43). The ability to modulate the immune responses has been reported for several skin AMPs, and it could be argued that reduced concentrations of AMPs on the skin after birth increase the risk for neonatal infections. However, the concentrations of AMPs in preterm infants differ not from those derived from healthy full-term newborns. As preterm infants have a 3–10 times higher incidence of infection than full-term infants (44), our observation cannot fully explain the increased risk for preterm infants. Taking our findings into account, the risk for sepsis should be nearly similar in both cohorts. Therefore, other immune-specific factors will play a role in the increase of infection risk in neonates.

In our cohort, the skin of premature infants is regularly cared for with sunflower oil. We therefore let out skin care on the left thigh to control for skin care effects. We here found no differences in the expression of psoriasin or RNase 7 in dependence of use of sunflower oil. Phototherapy did not influence the expression of AMPs (data not shown).

Our study has some limitations. As this study was initially planned as explorative study, the first 13 participants were only analyzed on day one and missing on the later examination dates. Furthermore, the explorative design does not allow to adjust for all different clinical parameters that potentially affect AMP expression. For example, the role of chorioamnionitis could not be classified in more detail in our analysis, as we did not record detailed placental pathology findings, e.g., Blanc-defined stages. Further analyses should take this information into account.

We analyzed the amount of psoriasin and RNase 7 as these AMPs are the most abundant ones found on human skin that serve immunomodulatory roles in skin immunity. The methods of collecting and analyzing skin rinsing probes is well-established (45). Different techniques for AMP sampling exist, which may hamper direct comparison between studies. Most studies use analysis of skin biopsies, tape strips, and rinsing solution from the skin. Rinsing solution techniques have the advantage as a minimally invasive technique and its possibility for multiple testing of the same patient over time. The use of skin biopsies has been predominant but has the disadvantage of being invasive and contains the risk of catching deeper layers of the skin with AMPs from other cells rather than AMPs locally present (46).

In conclusion, this study shows that with increasing age of infants, concentrations of skin derived AMPs rise and that infectious complications modify the expression of psoriasin and RNase 7. Further studies are needed to clarify if varying concentrations of skin AMPs are dependent from maturing skin structures or developing microbial colonization and if AMP concentrations impact the risk for infections in newborns.

## Data availability statement

The raw data supporting the conclusions of this article will be made available by the authors, without undue reservation.

## Ethics statement

The studies involving human participants were reviewed and approved by the local ethics committee for research in human subjects of the University of Lübeck (file number 19-028). Written informed consent to participate in this study was provided by the participants’ legal guardian/next of kin.

## Author contributions

Conceived and designed the experiments: AH, LN, HB, HH, JH, and CH. Performed the experiments: LN, HH, JH, and HB. Analyzed the data: AH, JH, MF, FR, and HH. Contributed reagents/materials/analysis tools: AH, HH, JH, MF, and EH. Wrote the paper: AH, JH, CH, and EH. The authors gave the final approval of the version to be published. Each author participated sufficiently in the work to take public responsibility for appropriate portions of the content and agreed to be accountable for all aspects of the work in ensuring that questions related to the accuracy or integrity of any part of the work are appropriately investigated and resolved. All authors contributed to the article and approved the submitted version.
